# Supplementary material for: Effects of virtual reality exercise on fatigue, pain, and psychological status among cancer patients: a meta-analysis
Source: PeerJ. 2025 Oct 29;13:e20196. doi: 10.7717/peerj.20196 (PMC12579480; doi:10.7717/peerj.20196)
Supplement: Supplemental Information 3 [file peerj-13-20196-s003.docx]

**Supplementary Material 1**

Search strategy

| Databases | Search strategy | Result  (Approximately) |
| --- | --- | --- |
| Scopus | #1: Title-Abs-Key (Virtual Reality)  #2: Title-Abs-Key (Exercise or Training)  #3: Title-Abs-Key (Cancer)  #5: #1 and #2 and #3  Search strings: TITLE-ABS-KEY("Virtual Reality") AND TITLE-ABS-KEY(Exercise or Training) AND TITLE-ABS-KEY(Cancer)  Limiters - Published Date: 20150101-20250418 | 39,625  1,132,714  1,943,733  205 |
| Pubmed | #1: [Title/Abstract] Virtual Reality  #2: [Title/Abstract] Exercise or Training  #3: [Title/Abstract] Cancer  #4: #1 and #2 and #3  Search strings: ((Virtual Reality[Title/Abstract]) AND (Exercise[Title/Abstract] OR Training[Title/Abstract])) AND (Cancer[Title/Abstract])  Filters: Publication date from 2015/01/01 to 2025/04/18 | 18,098  641,675  1,333,383  116 |
| Web of Science | #1: TOPIC: (Virtual Reality)  #2: TOPIC: (Exercise or Training)  #3: TOPIC: (Cancer)  #4: #1 and #2 and #3  Search strings: Virtual Reality (Topic) and Exercise or Training (Topic) and Cancer (Topic)  Refined by: PUBLICATION YEARS: (20250418-20150101)  Indexes=SCI-EXPANDED, SSCI, CCR-EXPANDED, | 36,511  938,847  1,804,503  181 |
| EBSCO | #1: Abstract: (Virtual Reality)  #2: Abstract: (Exercise or Training)  #3: Abstract: (Cancer)  #4: #1 and #2 and #3  Search strings: AB “Virtual Reality” AND AB (Exercise or Training) AND AB Cancer  Year: 20150101-20250418 | 26,712  749,628  1,647,132  144 |
