# Supplementary material for: Effects of virtual reality exercise on fatigue, pain, and psychological status among cancer patients: a meta-analysis
Source: PeerJ. 2025 Oct 29;13:e20196. doi: 10.7717/peerj.20196 (PMC12579480; doi:10.7717/peerj.20196)
Supplement: Supplemental Information 4 [file peerj-13-20196-s004.docx]

Cancer is an important health care problem. Cancer and its related therapies may lead to psychological depression with pain, fatigue and high anxiety. This study provides an intervention program with low side effects in order to improve the fatigue and pain levels of cancer patients. For cancer patients and oncology related physicians who want to improve the quality of life, it provides a diversified choice of non-drug intervention programs.
